# Strong Cumulative Evidence of Associations of 6 Single Nucleotide Polymorphisms with Ovarian Cancer Risk: An Umbrella Review

**DOI:** 10.3390/jcm12052025

**Published:** 2023-03-03

**Authors:** Ying-Jun Huo, Xiao-Ying Li, Meng Zhang, Chang Gao, Qian Xiao, Yu-Hong Zhao, Song Gao, Ting-Ting Gong, Qi-Jun Wu

**Affiliations:** 1 Department of Clinical Epidemiology, Shengjing Hospital of China Medical University, No. 36, San Hao Street, Shenyang 110004, China; 2 Clinical Research Center, Shengjing Hospital of China Medical University, Shenyang 110004, China; 3 Key Laboratory of Precision Medical Research on Major Chronic Disease, Shenyang 110004, China; 4 Department of Obstetrics and Gynecology, Shengjing Hospital of China Medical University, No. 36, San Hao Street, Shenyang 110004, China

**Keywords:** association, meta-analysis, ovarian cancer, risk, single nucleotide polymorphism, umbrella review

## Abstract

**Background**: An increasing number of studies have reported associations between single nucleotide polymorphisms (SNPs) and ovarian cancer (OC) risk. However, some of the findings were inconsistent. The objective of this umbrella review was to evaluate the associations comprehensively and quantitatively. **Methods:** The protocol of this review was registered in PROSPERO (No. CRD42022332222). We searched the PubMed, Web of Science, and Embase databases to identify related systematic reviews and meta-analyses from inception to 15 October 2021. In addition to estimating the summary effect size by using fixed and random effects models and calculating the 95% prediction interval, we evaluated the cumulative evidence for associations with nominally statistical significance based on the Venice criteria and false positive report probability (FPRP). **Results**: Forty articles were included in this umbrella review, which referred to a total of 54 SNPs. The median number of original studies per meta-analysis was four, while the median number of total subjects was 3455. All included articles had greater than moderate methodological quality. A total of 18 SNPs were nominally statistically associated with OC risk; 6 SNPs (8 genetic models), 5 SNPs (7 genetic models), and 16 SNPs (25 genetic models) were identified as strong, moderate, and weak cumulative evidence, respectively. **Conclusion**: This umbrella review revealed associations between SNPs and OC risk and suggested strong cumulative evidence of associations of six SNPs (eight genetic models) with OC risk.

## 1. Introduction

Ovarian cancer (OC) is one of the most common gynecologic cancers and has become the leading cause of death from gynecologic cancers [1]. About 310,000 new cases and 210,000 deaths of OC worldwide occurred in 2020 [2]. The pathogenesis of OC is a complex multi-stage process involving genetic and non-genetic factors [3,4]. Increasing molecular epidemiological and experimental studies have demonstrated that genetic variations play important roles in the occurrence of OC [3].

The nucleotide variation with the frequency greater than 1% in a population, which is named as the single nucleotide polymorphism (SNP), is the most common genetic variation type in the human genome [5,6]. There has been significant progress in identifying common risk variants for OC using genome-wide association studies. Thirty-nine independent variant susceptibility regions of the epithelial OC have been summarized for future clinical risk prediction and prevention [7]. Moreover, a growing number of original studies, systematic reviews, and meta-analyses have investigated the associations between SNPs and OC risk during the recent years.

Tanha K. et al. performed an umbrella review based on systematic reviews and meta-analyses to identify factors associated with OC risk [8]. However, they only carried out data synthesis and basic statistical analysis to evaluate the associations between certain factors and OC risk. Of note, only a tiny fraction of these factors were SNPs. Up to now, there has not been a targeted umbrella review that extracts data from systematic reviews and meta-analyses to comprehensively and accurately evaluate the associations between SNPs and OC risk. Recently, a well-defined assessment framework—Venice criteria—has been specifically developed for genetic data, and the credibility of associations is assessed for three criteria: the amount of evidence, the extent of replication and protection from bias [9]. The criteria were widely used in assessing existing evidence as it improved the consistency and objectiveness of the genetic associations’ interpretation [8,10]. The false-positive report probability (FPRP), defined as the probability of no association given a statistically significant finding, is an effective approach to verify the noteworthiness of significant findings [11]. If the FPRP value of the significant association between SNP and OC risk was lower than the threshold, this would suggest that the association was noteworthy and authentic. Therefore, based on the Venice criteria and FPRP, this umbrella review was undertaken to comprehensively assess the credibility and strength of significant associations between SNPs and OC risk and highlight associations with the strongest epidemiological evidence.

## 2. Methods

An umbrella review is an approach that conducts a systematic and comprehensive survey and evaluation of systematic reviews and meta-analyses on a specific topic [12]. We performed an umbrella review based on the Preferred Reporting Items for Reviews and Meta-Analysis and Meta-analyses of Observational Studies in Epidemiology [13,14]. This umbrella review was registered in the PROSPERO, an international prospective register of systematic reviews, with the registration number CRD 42022332222.

### 2.1. Literature Search Strategy

To identify relevant systematic reviews and meta-analyses, we searched records in the PubMed, Embase, and Web of Science databases until 15 October 2021 with a comprehensive search strategy (Supplementary File S1). Additionally, we manually checked references of retrieved articles to avoid omissions.

### 2.2. Eligibility Criteria and Data Extraction

Articles with the following characteristics were included: (1) systematic reviews or meta-analyses on associations between SNPs and OC risk; (2) with quantitative synthesis; (3) limited to observational studies; (4) providing the number of cases and controls/population participants of all included studies; (5) showing genotyping data or risk estimates with 95% confidence interval (CI) of all included studies; and (6) written in English.

Articles with the following characteristics were excluded: (1) including family-based studies; (2) including studies with non-human subjects or without cancer-free controls; (3) investigating variants with ranges greater than one SNP; (4) evaluating the diagnosis, survival, or recurrence of OC; (5) systematic reviews or meta-analyses based on individual data; (6) conference abstracts, editorial comments, letters to editors, or unpublished literature; or (7) with fewer than three included studies.

If an eligible systematic review or meta-analysis was based on more than one SNP, each SNP was evaluated separately. For the same SNP, if there was more than one eligible systematic review or meta-analysis, the most recently published one was retained (the time was subject to the deadline of literature inclusion). Moreover, most of the eligible systematic reviews or meta-analyses referred to multiple races. Eligible articles were screened by two independent authors (Y.-J.H. and M.Z.), and a third author (X.-Y.L.) resolved inconsistencies.

For each included systematic review or meta-analysis, the following items were extracted: (1) the first author’s name; (2) year of publication; (3) target SNP; (4) gene name; (5) the number of included studies; (6) the number of cases and controls/population participants of each included study; (7) type of study design (e.g., case-control study) for each included study; (8) genotyping data or risk estimates with 95% CI of each included study (genotyping data were preferred); and (9) results of the Hardy-Weinberg equilibrium (HWE) test of each included study. Two independent authors (Y.-J.H. and M.Z.) extracted data from each eligible article, and disagreements were resolved by the third author (X.-Y.L.).

### 2.3. Methodological Quality Assessment for Included Articles

Two independent authors (Y.-J.H. and M.Z.) evaluated the methodological quality of all included articles based on the Assessment of Multiple Systematic Reviews (AMSTAR) tool, and the third author (X.-Y.L.) resolved inconsistencies [15]. This tool is an 11-item questionnaire requiring assessors to answer “yes”, “no”, “cannot answer”, or “not applicable”, and each item is scored as 1 point for “yes” or 0 points for other answers. The article quality was assessed as high, moderate, or low, with a total score of AMSTAR ≥ 8, 4–7, or ≤3 [16].

## 3. Statistical Analysis

### 3.1. Genetic Model Analysis

If the HWE results of included studies were unattainable, the chi square test was used to evaluate the HWE. Due to the lack of an optimal genetic model, five common genetic models were used for analysis in this umbrella review, unless the corresponding data for some genetic models were not available. For example, if a SNP was G/C, then five genetic models were: (1) the heterozygote comparison model (GC vs. GG); (2) the homozygote comparison model (CC vs. GG); (3) the dominant model (GC+CC vs. GG); (4) the recessive model (CC vs. GG+GC); and (5) the allele model (C vs. G).

### 3.2. Assessment of Heterogeneity and Pooled Effects

Fixed-effects models and random-effects models were applied to calculate the pooled effects and 95% CI for each meta-analysis [17,18]. Considering the conservativeness, the major consequence was based on the random-effects models, and *p* < 0.05 was thought to be nominally statistically significant [19]. For the summary random effects, the 95% prediction interval (PI) was calculated to account for between-study heterogeneity and estimate the uncertainty of the effect that would be expected if another study researched the same association [20,21]. The heterogeneity among studies was evaluated using the *I*^2^ statistic [22]. *I*^2^ > 50% indicated the large between-study heterogeneity. The 95% CI of *I*^2^ was calculated according to the method of Ioannidis et al. [23].

### 3.3. Evaluation of Bias

For SNPs with nominal statistical significance, potential bias was further evaluated. Firstly, we calculated the *p* value of the Egger’s asymmetry test as an indication of small-study effects [24]. Small-study effects existed when *p* value was less than 0.10 and when there was a more conservative effect size in the largest study than in those from random-effects meta-analysis [25]. Secondly, we compared the observed number of studies (O) of nominally significant results included in a meta-analysis with the expected number (E) of statistically significant results, by using the excess significance test [26]. The excess significance test was considered positive when *p* < 0.10 and O > E [26]. Third, we evaluated whether the nominally statistical significance was lost after excluding the first published study [19]. Fourth, we evaluated whether the nominally statistical significance was lost after excluding studies that violated the HWE (*p* < 0.05) [27]. All analyses were two-sided and performed by Stata 11 software (Stata LLC, College Station, TX, USA).

### 3.4. Evaluation of Cumulative Evidence

**Figure 1:**
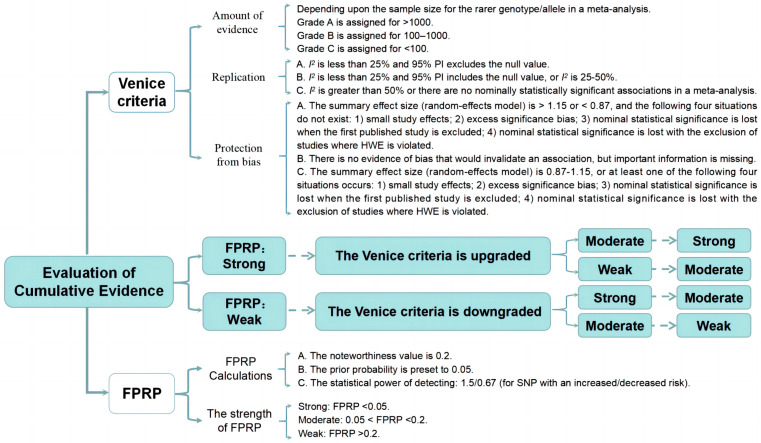
The criterion for evaluating cumulative evidence.

We further assessed the cumulative evidence of SNPs with nominally statistical significance. First, Venice criteria were used to assess the strength of evidence indicating epidemiological credibility [9]. Venice criteria have been widely used in similar studies, including three items: the amount of evidence, replication, and protection from bias [19,28,29,30]. These items were rated as A, B, or C, as described in Figure 1. Minor allele frequency (MAF) is the frequency of alleles that are rarely in each population. If the sample size of the rarer allele in a meta-analysis was unavailable, it was calculated based on the MAF, which was retrieved from the SNP database of the National Center for Biotechnology Information (https://www.ncbi.nlm.nih.gov/snp/, Last visit: 27 April 2022).

Further, the Excel spreadsheet on the Wacholder website was used to assess FPRP [11]. Wacholder et al. suggested the prior probability was preset as 0.05, the FPRP noteworthiness value was set as 0.2, and the statistical power was set to identify an OR of 0.67 (for SNP with a protective effect) or an OR of 1.5 (for SNP with an dangerous effect) [11]. The strength of FPRP was divided into three grades: strong (FPRP < 0.05), moderate (0.05 < FPRP < 0.2), and weak (FPRP > 0.2). We further combined the Venice criteria and FPRP to accurately evaluate the cumulative evidence. If the FPRP was evaluated as strong, we upgraded the evidence strength determined by the Venice criteria from weak to moderate or from moderate to strong. Otherwise, if the FPRP was evaluated as weak, we downgraded the evidence strength determined by the Venice criteria from moderate to weak or from strong to moderate [31]. A sensitivity analysis was given when SNP was ultimately rated as “strong” by evaluation of cumulative evidence.

## 4. Results

### 4.1. Overall Characteristics

**Figure 2:**
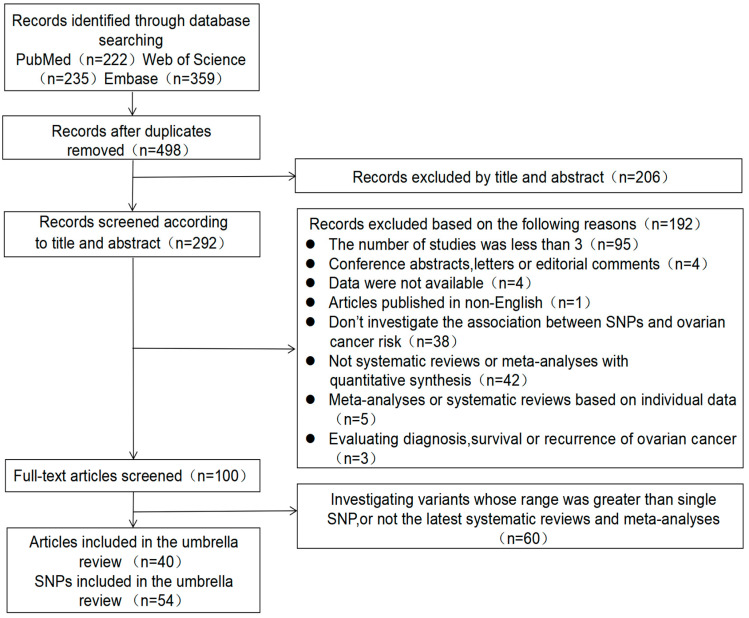
The screening process of articles.

We initially identified 222 records from PubMed, 359 records from Embase, and 235 records from the Web of Science. After accounting for the duplication and the criteria of inclusion and exclusion, the final 40 articles were included for this umbrella review, which referred to a total of 54 SNPs (Figure 2).

Supplementary File S2 shows the basic characteristics of the 40 included articles, which were published between 2010 and 2021. More than half of the included articles (*n* = 25, 62.5%) were published after 2017. In these systematic reviews or meta-analyses, all original studies were case-control studies, including subjects from multiple countries worldwide. No GWAS study was involved in these case-control studies. All included articles had greater than moderate quality, which was evaluated according to AMSTAR score. 

The median number of original studies per meta-analysis was 4 (ranging from 3 to 24), while the median number of cases, controls, and total subjects were 1375 (ranging from 143 to 6338), 2056 (ranging from 246 to 10,496), and 3455 (ranging from 394 to 16,834), respectively (Table 1 and Supplementary File S3). All subjects were OC patients or cancer-free individuals.

### 4.2. The Cumulative Evidence Evaluation of Associations between SNPs and OC Risk

**Figure 3:**
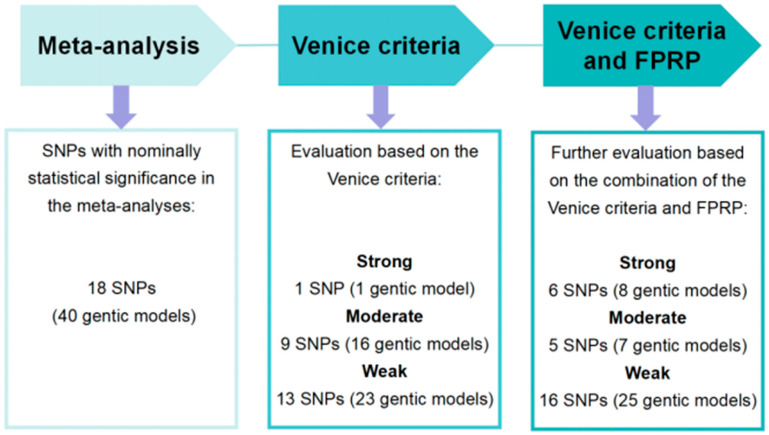
The process of the assessment for cumulative evidence.

Of the 54 SNPs identified, only 18 SNPs were nominally statistically associated with OC risk in at least one genetic model and were located on 13 genes and one miRNA. Only rs2228570 (model 2) of the vitamin D receptor (VDR) was rated as strong evidence according to the Venice criteria. Moreover, 16 genetic models of 9 SNPs and 23 genetic models of 13 SNPs were rated as moderate and weak evidence, respectively (Table 2 and Figure 3).

We further evaluated the cumulative evidence on the associations between SNPs and OC risk based on the combination of the Venice criteria and FPRP (Table 2 and Figure 3). A total of six SNPs (eight genetic models) were identified as strong cumulative evidence, including rs3020450, rs11614913, rs28362491, rs1052133, rs2228570, and rs833061. Meanwhile, a total of 5 SNPs (7 genetic models) were identified as moderate cumulative evidence and 16 SNPs (25 genetic models) were identified as weak cumulative evidence. Additionally, there were 36 SNPs without nominally statistical association with OC risk in any genetic model (Supplementary File S3).

## 5. Discussion

Our study is the first umbrella review to estimate the existing evidence of the relationships between SNPs and OC risk comprehensively and objectively. Our umbrella review identified the cumulative evidence on associations between OC risk and 6 SNPs (8 genetic models), 5 SNPs (7 genetic models), and 16 SNPs (25 genetic models) as strong, moderate, and weak, respectively. Notably, six SNPs (rs3020450, rs11614913, rs28362491, rs1052133, rs2228570, and rs833061) with strong cumulative evidence were located on *ESR2*, miR-196a2, *NFKB1*, *OGG1*, *VDR*, and *VEGFA*, respectively.

Discrepancies between the study of Tanha K et al. and our umbrella review regarding the contribution of SNPs to OC risk are most likely explained by methodological differences [8]. Tanha K et al. only carried out data synthesis and Egger’s regression asymmetry tests to identify factors associated with OC risk [8]. In contrast, we not only focused on SNPs but also further assessed cumulative evidence between SNPs and OC risk using the Venice criteria and FPRP tests. We provided a robust synthesis of published articles and increased the conclusive power with precise estimates. 

The *ESR2* (estrogen receptor 2) gene, also known as *Erb*, *ESRB*, *ODG8*, *ESTRB*, *NR3A2*, *ER-BETA,* and *ESR-BETA*, is usually described as a tumor suppressor. *ESR2* can regulate genes in several key pathways, such as tumor suppression, survival, metabolism, and proliferation pathways [32]. In the normal ovary, *ER-BETA* was shown to be capable of enhancing FasL expression, a major apoptotic regulator [33]. SNP rs3020450 of the *ESR2* gene is located at 14q23.2-q23.3 and belongs to a genic upstream transcript variant or an intron variant. Mutation Taster (https://www.mutationtaster.org/, Last visit: 22 February 2023), is a popular website for predicting the functional impairment of proteins caused by mutations [34]. According to the prediction of Mutation Taster, rs3020450 might cause poly(A) signal changes and splice site changes and might not be conserved in the species. In this study, the heterozygote comparison model and the dominant model of rs3020450 fit in the strong evidence class. Compared with the GG genotype, the GA genotype and the GA + AA genotype were associated with a reduced risk of OC.

MiR-196a2 (microRNA 196a-2) is an endogenous non-coding RNAs with regulatory functions, which may play a vital role in the development and progression of OC [35,36]. SNP rs11614913 of miR-196a2 at 12q13.13 belongs to a non-coding transcript variant. Results from a series of functional experiments suggested that elevated miR-196a expression from C allele-transfected cells promoted cell proliferation, migration, and invasive capacity in vitro [37]. In this umbrella review, the recessive models of rs11614913 showed a highly significant association with OC risk. Compared with the CC + CT genotype, the TT genotype was associated with a reduced risk of OC. 

*NFKB1* (nuclear factor kappa B sub unit 1) gene, also known as *CVID12*, *EBP-1*, *KBF1*, *NF-kB*, *NF-kB1*, *NF-kappa-B1*, *NF-kappa B*, *NF-kappa beta*, *NFKB-p105*, *NFKB-p50,* and *NFkappaB*, has been identified as a haploid insufficient DNA damage-specific tumor suppressor [38]. A previous study demonstrated that miR-9 could inhibit OC cell growth through regulating *NF-kappaB1* [39]. SNP rs28362491, namely the -94insertion/deletion(I/D) ATTG polymorphism, is localized in the 2kb upstream of the *NFKB1* gene at 4q24. The I allele of rs28362491 was proven to increase transcriptional activity, and the expression of p50 with tumor-promoting effect was up-regulated [40]. Mutation Taster predicted that rs28362491 was pathogenic with a probability of 1 and was highly conserved in the species. Our study provided strong evidence for the association between rs28362491 and OC risk via the heterozygote comparison model and the dominant model. In contrast to the II genotype, the ID and ID + DD genotypes were associated with a reduced risk of OC. A meta-analysis supported our result that D allele of rs28362491 was a protective allele for susceptibility to cancer [41].

The *OGG1* (8-oxoguanine DNA glycosylase) gene, also known as *HMMH*, *HOGG1*, *MUTM,* and *OGH1,* encodes the DNA repair enzyme and thus plays an important role in the development and progression of tumors [42]. HOGG1 can excise and remove 8-oxoguanine adducts from damaged DNA to play a repairing role [43]. SNP rs1052133 (Ser326Cys) of the *OGG1* is at position 1245 in exon 7. Based on the prediction of Mutation Taster, rs1052133 might not be conserved in the species and might cause the poly (A) signal changes and splice site changes. In this umbrella review, relationships between rs1052133 and OC risk had strong cumulative evidence in the heterozygote comparison model. The CG genotype was associated with a lower risk of OC compared with that of the CC genotype.

The *VDR* (vitamin D receptor) gene, also known as *NR1I1* and *PPP1R163*, encodes the vitamin D3 receptor, which regulates a variety of metabolic pathways and thus has anti-cancer effects [44]. SNP rs2228570 of the *VDR* gene is located at 12q13.11 and is an initiator codon variant [45]. Mutation Taster predicted that rs2228570 might lead to splice site changes and might be conserved in the species. Our umbrella review found that rs2228570 and OC risk had strong cumulative evidence in the homozygote comparison model. Carriers of the TT genotype were at increased risk of OC compared with women with the CC genotype. A pooled analysis of five studies within the Ovarian Cancer Association Consortium provided evidence that VDR rs2228570 polymorphism might influence OC susceptibility. Its results indicated the carriers of the rare T allele were at increased risk of OC compared with women with the CC genotype [46]. The result was in line with our study.

The *VEGFA* (vascular endothelial growth factor A) gene, also known as *MVCD1*, *VEGF*, and *VPF*, is a member of the *PDGF/VEGF* growth factor family. *VEGFA* widely expresses in nearly all cancers and is recognized as the most crucial tumor angiogenesis factor [47]. SNP rs833061 is localized to the 2kb upstream of the *VEGFA* gene at 6p21.1. A previous study observed that the C allele was associated with increased *VEGF* promoter activity. That is, the C allele of rs833061 might enhance tumor angiogenesis [48]. In addition, the Mutation Taster tool suggested that rs833061 was a “disease causing” variant, and it was highly conserved in the species. In our umbrella review, rs833061 was associated with susceptibility to OC, as suggested by the strong cumulative evidence in the dominant model. The CT + TT genotype was associated with a higher risk of OC compared with the CC genotype.

SNP-related research is of great significance in the OC risk prediction. Researchers can use single nucleotide polymorphism networks, combined with certain computer algorithms, to make risk predictions for multiple diseases, including OC [49,50,51]. A portion of the genetic risk for complex diseases might stem from the interactive effect of multiple SNPs with low or modest risk. Combining the weighted values of these variants results in the generation of a polygenic risk score (PRS) [52]. PRS is of great significance in various aspects, such as for informing population screening programs, facilitating diagnoses, and predicting prognostic outcomes [53]. For example, Dareng EO et al. built a polygenic risk model for the prediction of the epithelial OC risk, based on PRS [54].

To date, there has not been an umbrella review that comprehensively assesses the association of SNPs with OC risk. Fundamentally, our study had more rigorous evaluation criteria than the study of Tanha K et al. [8]. In this study, we combined Venice criteria and FPRP in order to further increase reliability of the findings. Moreover, we searched three databases through a comprehensive strategy, and two authors independently extracted the information. Furthermore, we used the AMSTAR criteria to evaluate the methodological quality, in addition most of the investigated meta-analyses achieving an ideal quality score.

Nevertheless, some limitations of our study still exist. First, although the efforts to systematically search the publications through three databases were performed, some publications were unobtainable, such as gray literature. This could limit the breadth of our results. However, these unobtainable publications only accounted for a minority of the whole publications and had little effect on the overall results. Second, errors or confusion of the major allele and the minor allele in the original studies could exist. However, we have kept in concordance with included systematic reviews or meta-analyses when defining the major allele and the minor allele, for the sake of minimizing the occurrence of mistakes. Third, the paucity of data did not allow us to assess gene–gene or gene–environment interactions. That is, there are no systematic reviews or meta-analyses of interaction analyses as well as the polygenic model for OC, and thus it was difficult to conduct an umbrella review. Last, we might have missed possible associations of certain SNPs that have been published but have not yet been assessed through meta-analyses. This is also the limitation of the methodology of umbrella reviews.

## 6. Conclusions

In conclusion, this umbrella review assessed the associations between SNPs and OC risk by combining the Venice Criteria and the FPRP test, and it found strong cumulative evidence of associations of six SNPs (eight genetic models) with OC risk. Collectively, our study provided the referenced information for further investigation into the genetic susceptibility of OC.

## Figures and Tables

**Table 1:** Meta-analysis results of SNPs with nominal statistical significance.

SNPs	Gene Name	Variant	Genetic Model	No. of Studies	Sample Size	*I^2^* (95% CI)	Summary Effect Size (95% CI)		*p* (R)	95%PI
							Fixed Effects	Random Effects		
rs1048943	*CYP1A1*	1 Ile; 2 Val	2	8	3066	33.5 (0, 71)	2.84 (1.73, 4.67)	2.57 (1.28, 5.14)	0.008	0.52, 12.62
-	-	-	4	8	3066	0.0 (0, 68)	2.35 (1.46, 3.76)	2.35 (1.46, 3.76)	<0.001	1.30, 4.23
rs3731249	*CDKN2A*	1A; 2T	1	3	11,172	0.0 (0, 90)	0.77 (0.64, 0.94)	0.77 (0.64, 0.94)	0.010	0.22, 2.73
-	-	-	3	3	11,186	0.0 (0, 90)	0.79 (0.65, 0.95)	0.79 (0.65, 0.95)	0.013	0.23, 2.72
-	-	-	5	3	22,372	0.0 (0, 90)	0.80 (0.67, 0.96)	0.80 (0.67, 0.96)	0.019	0.24, 2.67
rs11515	*CDKN2A*	1C; 2G	2	3	5174	0.0 (0, 90)	1.60 (1.13, 2.28)	1.60 (1.13, 2.28)	0.009	0.16, 15.65
-	-	-	4	3	6773	0.0 (0, 90)	1.62 (1.14, 2.31)	1.62 (1.14, 2.31)	0.007	0.17, 15.75
rs1271572	*ESR2*	1G; 2T	4	8	12,002	23.8 (0, 65)	1.14 (1.04, 1.24)	1.15 (1.02, 1.29)	0.018	0.90, 1.46
rs3020450	*ESR2*	1G; 2A	1	4	2035	0.0 (0, 85)	0.72 (0.60, 0.86)	0.72 (0.60, 0.86)	<0.001	0.48, 1.07
-	-	-	3	4	2210	0.0 (0, 85)	0.74 (0.62, 0.87)	0.74 (0.62, 0.87)	<0.001	0.51, 1.07
-	-	-	5	4	4420	10.6 (0, 86)	0.82 (0.72, 0.94)	0.83 (0.71, 0.97)	0.017	0.55, 1.25
rs13181	*ERCC2*	1A; 2C	2	9	2406	77.6 (58, 88)	2.50 (1.95, 3.21)	2.12 (1.14, 3.97)	0.018	0.30, 15.20
-	-	-	3	9	4024	49.8 (0, 77)	1.39 (1.17, 1.65)	1.44 (1.11, 1.86)	0.006	0.71, 2.89
rs1800871	*IL10*	1C; 2T	1	3	799	0.0 (0, 90)	1.61 (1.10, 2.35)	1.61 (1.10, 2.35)	0.015	0.13, 19.13
rs1466445	*ITGA1*	19A; 26A	5	4	5878	0.0 (0, 85)	1.25 (1.02, 1.52)	1.25 (1.02, 1.52)	0.030	0.81, 1.93
rs11614913	miR-196a2	1C; 2T	4	4	2009	0.0 (0, 85)	0.73 (0.60, 0.89)	0.73 (0.60, 0.89)	0.002	0.47, 1.13
rs28362491	*NFKB1*	1I; 2D	1	4	2215	0.0 (0, 85)	0.73 (0.61, 0.87)	0.73 (0.61, 0.87)	0.001	0.49, 1.08
-	-	-	2	4	1544	39.7 (0, 80)	0.57 (0.46, 0.71)	0.54 (0.40, 0.73)	<0.001	0.19, 1.53
-	-	-	3	4	3036	0.0 (0, 85)	0.67 (0.56, 0.79)	0.67 (0.56, 0.79)	<0.001	0.46, 0.97
-	-	-	4	4	3036	51.6 (0, 84)	0.73 (0.62, 0.87)	0.68 (0.52, 0.89)	0.005	0.24, 1.88
-	-	-	5	4	6072	38.5 (0, 79)	0.77 (0.69, 0.85)	0.75 (0.65, 0.86)	<0.001	0.46, 1.21
rs1052133	*OGG1*	1C; 2G	1	3	1836	0.0 (0, 90)	0.62 (0.51, 0.76)	0.62 (0.51, 0.76)	<0.001	0.17, 2.33
rs11466445	*TGFBR1*	19A; 26A	5	4	5878	0.0 (0, 85)	1.25 (1.02, 1.52)	1.25 (1.02, 1.52)	0.030	0.81, 1.93
rs2228570	*VDR*	1C; 2T	1	14	9952	26.6 (0, 61)	1.12 (1.03, 1.22)	1.12 (1.01, 1.25)	0.029	0.88, 1.43
-	-	-	2	14	6232	0.0 (0, 55)	1.18 (1.05, 1.33)	1.18 (1.05, 1.33)	0.005	1.04, 1.34
-	-	-	3	14	11,690	5.4 (0, 57)	1.13 (1.05, 1.23)	1.14 (1.04, 1.23)	0.003	1.00, 1.28
-	-	-	5	14	23,380	0.0 (0, 55)	1.09 (1.04, 1.16)	1.09 (1.04, 1.16)	0.001	1.03, 1.16
rs11568820	*VDR*	1G; 2A	1	3	3362	0.0 (0, 90)	1.21 (1.05, 1.41)	1.21 (1.05, 1.41)	0.011	0.47, 3.16
-	-	-	3	3	3530	0.0 (0, 90)	1.19 (1.04, 1.37)	1.19 (1.04, 1.37)	0.014	0.48, 2.98
-	-	-	5	3	7060	0.0 (0, 90)	1.13 (1.01, 1.28)	1.13 (1.01, 1.28)	0.039	0.53, 2.44
rs7975232	*VDR*	1a; 2A	5	4	980	0.0 (0, 85)	1.24 (1.02, 1.50)	1.24 (1.02, 1.50)	0.032	0.81, 1.89
rs833061	*VEGFA*	1C; 2T	2	4	1004	30.3 (0, 75)	2.48 (1.72, 3.58)	2.35 (1.49, 3.71)	<0.001	0.53, 10.46
-	-	-	3	4	1718	9.1 (0, 86)	1.96 (1.50, 2.57)	1.89 (1.40, 2.57)	<0.001	0.84, 4.26
-	-	-	4	4	1718	75.8 (33, 91)	1.68 (1.34, 2.10)	1.84 (1.13, 2.98)	0.014	0.22, 15.08
-	-	-	5	4	3436	67.8 (6, 89)	1.58 (1.36, 1.83)	1.54 (1.16, 2.03)	0.003	0.48, 4.96
rs3218536	*XRCC2*	1G; 2A	1	8	13,081	0.0 (0, 68)	0.87 (0.78, 0.97)	0.87 (0.78, 0.97)	0.012	0.76, 1.00
-	-	-	3	8	13,168	0.0 (0, 68)	0.86 (0.77, 0.95)	0.86 (0.77, 0.95)	0.004	0.75, 0.98
-	-	-	5	8	26,336	0.0 (0, 68)	0.85 (0.77, 0.94)	0.85 (0.77, 0.94)	0.001	0.75, 0.96
rs1799794	*XRCC3*	1A; 2G	2	4	4591	0.0 (0, 85)	0.72 (0.53, 0.97)	0.72 (0.53, 0.97)	0.029	0.37, 1.38
-	-	-	4	4	6689	0.0 (0, 85)	0.69 (0.51, 0.92)	0.69 (0.51, 0.92)	0.013	0.36, 1.31

CI: confidence interval; *p* (R): *p* value of the random effect; PI: prediction intervals; SNP: single nucleotide polymorphism.

**Table 2:** Cumulative evidence details of SNPs with nominal statistical significance.

SNPs	Genetic Model	P (Excluding the First Published Study)	P (Excluding Studies that Violated HWE)	Small-Study Effect	Excess Significance	Venice Criteria	*p* (FPRP)	Cumulative Evidence
Strong								
rs3020450	1	0.001	<0.001	NO	NO	Moderate (B/B/A)	0.007	Strong
-	3	<0.001	<0.001	NO	NO	Moderate (B/B/A)	0.010	Strong
rs11614913	4	0.002	0.002	NO	NO	Moderate (B/B/A)	0.045	Strong
rs28362491	1	0.004	0.001	NO	NO	Moderate (B/B/A)	0.013	Strong
-	3	<0.001	<0.001	NO	NO	Moderate (B/A/A)	<0.001	Strong
rs1052133	1	<0.001	<0.001	NO	NO	Moderate (B/B/A)	<0.001	Strong
rs2228570	2	0.007	0.005	NO	NO	Strong (A/A/A)	0.093	Strong
rs833061	3	<0.001	<0.001	NO	NO	Moderate (B/B/A)	0.011	Strong
Moderate								
rs1048943	4	<0.001	0.002	NO	NO	Moderate (B/A/A)	0.195	Moderate
rs28362491	2	<0.001	<0.001	YES	YES	Weak (B/B/C)	0.011	Moderate
-	5	0.001	<0.001	YES	YES	Weak (A/B/C)	<0.001	Moderate
rs2228570	3	0.005	0.003	NO	NO	Weak (A/A/C)	0.045	Moderate
-	5	0.003	0.001	NO	NO	Weak (A/A/C)	0.026	Moderate
rs11568820	1	0.009	0.011	NO	NO	Moderate (B/B/A)	0.175	Moderate
rs833061	2	0.001	0.018	NO	NO	Moderate (B/B/A)	0.146	Moderate
Weak								
rs1048943	2	<0.001	0.014	NO	NO	Moderate (B/B/A)	0.694	Weak
rs3731249	1	0.006	0.010	NO	YES	Weak (C/B/C)	0.167	Weak
-	3	0.009	0.013	NO	NO	Weak (C/B/A)	0.205	Weak
-	5	0.013	0.019	NO	NO	Moderate (B/B/A)	0.272	Weak
rs11515	2	0.096	0.009	NO	NO	Weak (B/B/C)	0.314	Weak
-	4	0.086	0.007	NO	NO	Weak (B/B/C)	0.280	Weak
rs1271572	4	0.016	0.020	YES	NO	Weak (A/B/C)	0.254	Weak
rs3020450	5	0.001	0.017	NO	NO	Moderate (A/B/A)	0.241	Weak
rs13181	2	0.106	0.043	NO	NO	Weak (A/C/C)	0.716	Weak
-	3	0.068	0.027	NO	NO	Weak (A/B/C)	0.151	Weak
rs1800871	1	0.106	0.259	NO	NO	Weak (NA/B/C)	0.442	Weak
rs1466445	5	0.077	0.030	NO	NO	Weak (B/B/C)	0.372	Weak
rs28362491	4	0.010	0.005	YES	YES	Weak (B/C/C)	0.144	Weak
rs11466445	5	0.154	0.030	NO	NO	Weak (B/B/C)	0.372	Weak
rs2228570	1	0.037	0.029	NO	NO	Weak (A/B/C)	0.352	Weak
rs11568820	3	0.015	0.014	NO	NO	Moderate (B/B/A)	0.214	Weak
-	5	0.050	0.039	NO	NO	Weak (A/B/C)	0.421	Weak
rs7975232	5	0.089	0.032	NO	NO	Weak (B */B/C)	0.380	Weak
rs833061	4	0.039	0.084	NO	NO	Weak (B/C/C)	0.555	Weak
-	5	0.008	0.058	NO	NO	Weak (A/C/C)	0.104	Weak
rs3218536	1	0.131	0.012	NO	NO	Weak (C/B/C)	0.193	Weak
-	3	0.082	0.004	NO	NO	Weak (C/A/C)	0.061	Weak
-	5	0.056	0.001	NO	NO	Weak (A/A/C)	0.074	Weak
rs1799794	2	0.023	0.029	NO	NO	Moderate (B/B/A)	0.452	Weak
-	4	0.011	0.013	NO	NO	Moderate (B/B/A)	0.298	Weak

NA: not available. * The sample size for the rarer allele in a meta-analysis was calculated based on the MAF offered by dbSNP of NCBI.

## Data Availability

Data are available upon request from the corresponding author.

## Supplementary Materials

The following supporting information can be downloaded at: https://www.mdpi.com/article/10.3390/jcm12052025/s1, File S1: Search strategy; File S2: Basic characteristics and quality assessment of all included articles; File S3: No nominally statistically significant associations in the meta-analyses.
