# Supplementary material for: Strong Cumulative Evidence of Associations of 6 Single Nucleotide Polymorphisms with Ovarian Cancer Risk: An Umbrella Review
Source: J Clin Med. 2023 Mar 3;12(5):2025. doi: 10.3390/jcm12052025 (PMC10004083; doi:10.3390/jcm12052025)
Supplement: Supplementary file 1 [file jcm-12-02025-s001.zip › jcm-2179460-supplementary/Supplementary File S1.pdf]

A) Search strategy for PubMed

1. “Polymorphism, Single Nucleotide”[Mesh]
2. Nucleotide Polymorphism, Single
3. Nucleotide Polymorphisms, Single
4. Polymorphisms, Single Nucleotide
5. Single Nucleotide Polymorphisms
6. SNPs
7. Single Nucleotide Polymorphism
8. #1 OR #2 OR #3 OR #4 OR #5 OR #6 OR #7
9. “Polymorphism, Genetic”[Mesh]
10. Polymorphisms, Genetic
11. Genetic Polymorphisms
12. Genetic Polymorphism
13. Polymorphism (Genetics)
14. Polymorphisms (Genetics)
15. #9 OR #10 OR #11 OR #12 OR #13 OR #14
16. #8 OR #15
17. “Ovarian Neoplasms”[Mesh]
18. Neoplasm, Ovarian
19. Ovarian Neoplasm
20. Ovary Neoplasms
21. Neoplasm, Ovary
22. Neoplasms, Ovary
23. Ovary Neoplasm
24. Neoplasms, Ovarian
25. Ovary Cancer
26. Cancer, Ovary
27. Cancers, Ovary
28. Ovary Cancers
29. Ovarian Cancer

30. Cancer, Ovarian

31. Cancers, Ovarian

32. Ovarian Cancers

33. Cancer of Ovary

34. Cancer of the Ovary

35. Ovarian Carcinoma

36. Ovarian Tumor

37. #17 OR #18 OR #19 OR #20 OR #21 OR #22 OR #23 OR #24 OR #25 OR #26 OR #27 OR #28 OR #29 OR #30 OR #31 OR #32 OR #33 OR #34 OR #35 OR #36 OR #36

38. Systematic Review

39. Meta-Analysis

40. #38 OR #39

41. #16 AND #37 AND #40

B) Search strategy for Web of Science

1. TS=(Polymorphism, Single Nucleotide)

2. TS=(Nucleotide Polymorphism, Single)

3. TS=(Nucleotide Polymorphisms, Single)

4. TS=(Polymorphisms, Single Nucleotide)

5. TS=(Single Nucleotide Polymorphisms)

6. TS=(SNPs)

7. TS=(Single Nucleotide Polymorphism)

8. #1 OR #2 OR #3 OR #4 OR #5 OR #6 OR #7

9. TS=(Polymorphism, Genetic)

10. TS=(Polymorphisms, Genetic)

11. TS=(Genetic Polymorphisms)

12. TS=(Genetic Polymorphism)

13. TS=(Polymorphism (Genetics))

14. TS=(Polymorphisms (Genetics))

15. #9 OR #10 OR #11 OR #12 OR #13 OR #14

16. #8 OR #15

17. TS=(Ovarian Neoplasms)

18. TS=(Neoplasm, Ovarian)

19. TS=(Ovarian Neoplasm)

20. TS=(Ovary Neoplasms)

21. TS=(Neoplasm, Ovary)

22. TS=(Neoplasms, Ovary)

23. TS=(Ovary Neoplasm)

24. TS=(Neoplasms, Ovarian)

25. TS=(Ovary Cancer)

26. TS=(Cancer, Ovary)

27. TS=(Cancers, Ovary)

28. TS=(Ovary Cancers)

29. TS=(Ovarian Cancer)

30. TS=(Cancer, Ovarian)

31. TS=(Cancers, Ovarian)

32. TS=(Ovarian Cancers)

33. TS=(Cancer of Ovary)

34. TS=(Cancer of the Ovary)

35. TS=(Ovarian Carcinoma)

36. TS=(Ovarian Tumor)

37. #17 OR #18 OR #19 OR #20 OR #21 OR #22 OR #23 OR #24 OR #25 OR #26 OR #27 OR #28 OR #29 OR #30 OR #31 OR #32 OR #33 OR #34 OR #35 OR #36

38. TS=(Systematic Review)

39. TS=(Meta-Analysis)

40. #38 OR #39

41. #16 AND #37 AND #40

C) Search strategy for Embase

1. ‘single nucleotide polymorphism’/exp
2. ‘polymorphism, single nucleotide’
3. ‘nucleotide polymorphism, single’
4. ‘nucleotide polymorphisms, single’
5. ‘polymorphisms, single nucleotide’
6. ‘single nucleotide polymorphisms’
7. ‘snps’
8. #1 OR #2 OR #3 OR #4 OR #5 OR #6 OR #7
9. ‘genetic polymorphism’/exp
10. ‘polymorphism, genetic’
11. ‘polymorphisms, genetic’
12. ‘genetic polymorphisms’
13. ‘polymorphism (genetics)’
14. ‘polymorphisms (genetics)’
15. #9 OR #10 OR #11 OR #12 OR #13 OR #14
16. #8 OR #15
17. ‘ovary cancer’/exp
18. ‘Ovarian Neoplasms’
19. ‘Neoplasm, Ovarian’
20. ‘Ovarian Neoplasm’
21. ‘Ovary Neoplasms’
22. ‘Neoplasm, Ovary’
23. ‘Neoplasms, Ovary’
24. ‘Ovary Neoplasm’
25. ‘Neoplasms, Ovarian’
26. ‘Cancer, Ovary’
27. ‘Cancers, Ovary’
28. ‘Ovary Cancers’
29. ‘Ovarian Cancer’
30. ‘Cancer, Ovarian’

31. ‘Cancers, Ovarian’
32. ‘Ovarian Cancers’
33. ‘Cancer of Ovary’
34. ‘Cancer of the Ovary’
35. ‘Ovarian Carcinoma’
36. ‘Ovarian Tumor’
37. #17 OR #18 OR #19 OR #20 OR #21 OR #22 OR #23 OR #24 OR #25 OR #26 OR #27 OR #28 OR #29 OR #30 OR #31 OR #32 OR #33 OR #34 OR #35 OR #36
38. ‘systematic review’
39. ‘meta-analysis’
40. #38 OR #39
41. #16 AND #37 AND #40
