# Supplementary material for: Strong Cumulative Evidence of Associations of 6 Single Nucleotide Polymorphisms with Ovarian Cancer Risk: An Umbrella Review
Source: J Clin Med. 2023 Mar 3;12(5):2025. doi: 10.3390/jcm12052025 (PMC10004083; doi:10.3390/jcm12052025)
Supplement: Supplementary file 1 [file jcm-12-02025-s001.zip › jcm-2179460-supplementary/Supplementary File S2.pdf]

Supplementary File S2. Basic characteristics and quality assessment of all included articles

The first author	Year of publication	SNPs	Region	AMSTAR score
Zhang et al.(1)	2021	rs1056836	America, China, France, Italy	6
Gajjar et al.(2)	2012	rs1056827, rs1800440, rs10012	America, France	7
Yuan et al.(3)	2020	rs3218536	Australia, America, Denmark, Egypt, England, Poland	7
Wang et al.(4)	2021	rs1801133, rs1801131	Australia, America, China, India, Poland, Turkey	7
Chen et al.(5)	2018	rs2228570	America, China, Denmark, England, Hawaii, Poland	8
Dai et al.(6)	2015	rs11568820	America	7
Wang et al.(7)	2013	rs1801320	Australia, Israel, Multiple, Poland	7
Tang et al.(8)	2018	rs1271572, rs1801200	Australia, America, Denmark, England, Finland, Germany, Iran, India, Japan, Philippines	8
Feng et al.(9)	2019	rs3020450	America, China, Germany, Japan, Philippines	8
Li et al.(10)	2017	rs144848	Australia, America, Czech, China, Denmark, England, Germany, Poland	7
Zhu et al.(11)	2017	rs1799750, rs34093618	America, China, Italy, Japan, Korea, Mexico, Poland	9
Peng et al.(12)	2010	rs35068180	China, Italy, Poland	7
Li et al.(13)	2017	rs13181	Brazil, China, Egypt, France, Poland, Portugal, Russia, Turkey	7
Yang et al.(14)	2019	rs11615	Asian	7
Zhang et al.(15)	2017	rs1042522	America, Australia, Brazil, China, Denmark, Greece, Israel, Japan, Morocco, New England, North India, Poland, Portugal, Roman, Serbia, South Africa	9
Xu et al.(16)	2017	rs3025039, rs833061, rs699947	Brazil, China, India, Poland, Turkey	8
Lu et al.(17)	2015	rs2066827	China, Egypt, England	7
Deng et al.(18)	2017	rs4045402	America, Australia, Italy	9
Liao et al.(19)	2015	rs10895068, rs1042838	America, Australia, France, Poland, The Netherlands	9
Shi et al.(20)	2020	rs1052133	China, Canada, India, Poland	9
Huang et al.(21)	2012	rs4646903, rs1048943	America, China, France, Germany, Japan, Poland, Turkish	9
Liu et al.(22)	2014	rs11466445	America, England, Poland	6
Jin et al.(23)	2019	rs1051740	America, Australia, China, England	6
Choupani et al.(24)	2019	rs11614913	China	7
Yu et al.(25)	2021	rs861539	Australia, Brazil, China, Mix, Polish	7

Liu et al.(26)	2021	rs1799794	America, England, Egypt, Mixed	9
Pan et al.(27)	2015	rs4680	America, France, Germany, Poland	7
Xu et al.(28)	2014	rs2273535	Australia, America, Denmark, Germany, England, Poland	4
Yu et al.(29)	2013	rs1800871	America, China, Germany	7
Dong et al.(30)	2017	rs3731249, rs11515, rs3088440	Australia, America, China, Denmark, England, Poland	6
Song et al.(31)	2013	rs7975232, rs731236, rs1544410	America, Sweden	9
Zhang et al.(32)	2016	rs61764370	American, Belarus, Belgium, Canada, Denmark, England, Finland, Germany, Holland	7
Zhao et al.(33)	2017	rs1695	Australia, Brazil, France	6
Tian et al.(34)	2020	rs1799793, rs238406	Brazil, China, France, Poland, Portugal, Russia	7
Fu et al.(35)	2017	rs28362491	China	7
Miao et al.(36)	2017	rs799917	America, Denmark, England	7
Wang et al.(37)	2015	rs1801282	American, England, Japan	7
Zhang et al.(38)	2018	rs2279744	China, Japan	6
Wang et al.(39)	2012	rs1466445	American, England, Poland	6
Zhou et al.(40)	2012	rs6917	Australia, Poland	6

AMSTAR, Assessment of Multiple Systematic Reviews; SNPs, single nucleotide polymorphisms.

References

1. Zhang L, Feng L, Lou M, Deng X, Liu C, Li L. The ovarian carcinoma risk with the polymorphisms of CYP1B1 come from the positive selection. American journal of translational research. 2021;13(5):4322-41.

2. Gajjar K, Owens G, Sperrin M, Martin-Hirsch PL, Martin FL. Cytochrome P1B1 (CYP1B1) polymorphisms and ovarian cancer risk: a meta-analysis. Toxicology. 2012;302(2-3):157-62.

3. Yuan C, Liu X, Li R, Yan S, Kong B. Analysis of the association between the XRCC2 rs3218536 polymorphism and ovarian cancer risk. Arch Med Sci. 2020;16(3):682-91.

4. Wang Z, Li K, Ouyang L, Iko H, Safi AJ, Gao S. Effects of methylenetetrahydrofolate reductase single-nucleotide polymorphisms on breast, cervical, ovarian, and endometrial cancer susceptibilities. Chronic Dis Transl Med. 2021;7(3):169-81.

5. Chen H, Zhu J. Vitamin D receptor rs2228570 polymorphism and susceptibility to ovarian cancer: An updated meta-analysis. J Obstet Gynaecol Res. 2018;44(3):556-65.

6. Dai ZM, Fei YL, Zhang WG, Liu J, Cao XM, Qu QM, et al. Association of Vitamin D Receptor Cdx-2 Polymorphism With Cancer Risk: A Meta-Analysis. Medicine (Baltimore). 2015;94(33):e1370.

7. Wang W, Li JL, He XF, Li AP, Cai YL, Xu N, et al. Association between the RAD51 135 G>C polymorphism and risk of cancer: a meta-analysis of 19,068 cases and 22,630 controls. PloS one. 2013;8(9):e75153.
8. Tang L, Li J, Bao M, Xiang J, Chen Y, Wang Y. Genetic association between HER2 and ESR2 polymorphisms and ovarian cancer: a meta-analysis. Onco Targets Ther. 2018;11:1055-66.
9. Feng Y, Peng Z, Liu W, Yang Z, Shang J, Cui L, et al. Evaluation of the epidemiological and prognosis significance of ESR2 rs3020450 polymorphism in ovarian cancer. Gene. 2019;710:316-23.
10. Li Q, Guan R, Qiao Y, Liu C, He N, Zhang X, et al. Association between the BRCA2 rs144848 polymorphism and cancer susceptibility: a meta-analysis. Oncotarget. 2017;8(24):39818-32.
11. Zhu XM, Sun WF. Association between matrix metalloproteinases polymorphisms and ovarian cancer risk: A meta-analysis and systematic review. PLoS One. 2017;12(9):e0185456.
12. Peng B, Cao L, Wang W, Xian L, Jiang D, Zhao J, et al. Polymorphisms in the promoter regions of matrix metalloproteinases 1 and 3 and cancer risk: a meta-analysis of 50 case-control studies. Mutagenesis. 2010;25(1):41-8.
13. Li J, Pan L, Qin X, Chu H, Mu H, Wan G. Association between ERCC2 rs13181 polymorphism and ovarian cancer risk: an updated meta-analysis with 4024 subjects. Arch Gynecol Obstet. 2017;296(3):551-8.
14. Yang F, Mu X, Bian C, Zhang H, Yi T, Zhao X, et al. Association of excision repair cross-complimentary group 1 gene polymorphisms with breast and ovarian cancer susceptibility. J Cell Biochem. 2019;120(9):15635-47.
15. Zhang A, Shi TY, Zhao Y, Xiang J, Yu D, Liang Z, et al. No association between TP53 Arg72Pro polymorphism and ovarian cancer risk: evidence from 10113 subjects. Oncotarget. 2017;8(68):112761-9.
16. Xu CH, He ZH, Xu H. Association of four genetic polymorphisms in the vascular endothelial growth factor-A gene and development of ovarian cancer: a meta-analysis. Oncotarget. 2017;8(42):73063-78.
17. Lu Y, Gao K, Zhang M, Zhou A, Zhou X, Guan Z, et al. Genetic Association Between CDKN1B rs2066827 Polymorphism and Susceptibility to Cancer. Medicine (Baltimore). 2015;94(46):e1217.
18. Deng Y, Wang J, Wang L, Du Y. Androgen receptor gene CAG repeat polymorphism and ovarian cancer risk: A meta-analysis. Biosci Trends. 2017;11(2):193-201.
19. Liao J, Ding D, Sun C, Weng D, Meng L, Chen G, et al. Polymorphisms of progesterone receptor and ovarian cancer risk: a systemic review and meta-analysis. J Obstet Gynaecol Res. 2015;41(2):178-87.
20. Shi Y, Xu W, Zhang X. Association of the hOGG1 Ser326Cys polymorphism with gynecologic cancer susceptibility: a meta-analysis. Biosci Rep. 2020;40(12).

21. Huang M, Chen Q, Xiao J, Zhao X, Liu C. CYP1A1 Ile462Val is a risk factor for ovarian cancer development. Cytokine. 2012;58(1):73-8.
22. Liu LL, Wei YP, Xu H, Huang Y, Luo FE, Huang ZJ, et al. A systematic review and meta-analysis of the association of transforming growth factor beta receptor I 6A/9A gene polymorphism with ovarian cancer risk. J Recept Signal Transduct Res. 2014;34(4):313-6.
23. Jin Y. Association between EPHX1 polymorphism rs1051740 and the risk of ovarian cancer: a meta-analysis. Artif Cells Nanomed Biotechnol. 2019;47(1):2338-42.
24. Choupani J, Nariman-Saleh-Fam Z, Saadatian Z, Ouladsahebmadarek E, Masotti A, Bastami M. Association of mir-196a-2 rs11614913 and mir-149 rs2292832 Polymorphisms With Risk of Cancer: An Updated Meta-Analysis. Front Genet. 2019;10:186.
25. Yu X, Wang Q, He G, Yu H. Association between XRCC3 Thr241Met polymorphism and risk of gynecological malignancies: A meta-analysis. Cancer Genet. 2021;254-255:11-7.
26. Liu W, Ma S, Liang L, Kou Z, Zhang H, Yang J. The association between XRCC3 rs1799794 polymorphism and cancer risk: a meta-analysis of 34 case-control studies. BMC Med Genomics. 2021;14(1):117.
27. Pan W, Liao H. Correlations between the COMT gene rs4680 polymorphism and susceptibility to ovarian cancer. Genet Mol Res. 2015;14(4):16813-8.
28. Xu L, Zhou X, Jiang F, Xu L, Yin R. STK15 rs2273535 polymorphism and cancer risk: a meta-analysis of 74,896 subjects. Cancer Epidemiol. 2014;38(2):111-7.
29. Yu Z, Liu Q, Huang C, Wu M, Li G. The interleukin 10 -819C/T polymorphism and cancer risk: a HuGE review and meta-analysis of 73 studies including 15,942 cases and 22,336 controls. OMICS. 2013;17(4):200-14.
30. Dong Y, Wang X, Yang YW, Liu YJ. The effects of CDKN2A rs3731249, rs11515, and rs3088440 polymorphisms on cancer risk. Cell Mol Biol (Noisy-le-grand). 2017;63(3):40-4.
31. Song GG, Lee YH. Vitamin D receptor FokI, BsmI, ApaI, and TaqI polymorphisms and susceptibility to ovarian cancer: a meta-analysis. Immunol Invest. 2013;42(7):661-72.
32. Zhang SY, Shi J. rs61764370 polymorphism of Kras and risk of cancer in Caucasian population: A meta-analysis. J Cancer Res Ther. 2016;12(2):699-704.
33. Zhao E, Hu K, Zhao Y. Associations of the glutathione S-transferase P1 Ile105Val genetic polymorphism with gynecological cancer susceptibility: a meta-analysis. Oncotarget. 2017;8(25):41734-9.
34. Tian Y, Lin X, Yang F, Zhao J, Yao K, Bian C. Contribution of xeroderma pigmentosum complementation group D gene polymorphisms in breast and ovarian cancer susceptibility: A protocol for systematic review and meta analysis. Medicine (Baltimore). 2020;99(21):e20299.
35. Fu W, Zhuo ZJ, Chen YC, Zhu J, Zhao Z, Jia W, et al. NFKB1 -94insertion/deletion ATTG polymorphism and cancer risk: Evidence from 50 case-control studies. Oncotarget. 2017;8(6):9806-22.

36. Miao L, Yu Y, Ji Y, Zhang B, Yuan Z, Du Y, et al. Association between BRCA1 P871L polymorphism and cancer risk: evidence from a meta-analysis. Oncotarget. 2017;8(18):30587-94.

37. Wang Y, Chen Y, Jiang H, Tang W, Kang M, Liu T, et al. Peroxisome proliferator-activated receptor gamma (PPARG) rs1801282 C>G polymorphism is associated with cancer susceptibility in asians: an updated meta-analysis. Int J Clin Exp Med. 2015;8(8):12661-73.

38. Zhang J, Zhang Y, Zhang Z. Association of rs2279744 and rs117039649 promoter polymorphism with the risk of gynecological cancer: A meta-analysis of case-control studies. Medicine (Baltimore). 2018;97(2):e9554.

39. Wang YQ, Qi XW, Wang F, Jiang J, Guo QN. Association between TGFBR1 polymorphisms and cancer risk: a meta-analysis of 35 case-control studies. PLoS One. 2012;7(8):e42899.

40. Zhou TB, Yin SS, Huang JJ, Ou C. Relationship between the prohibitin 3' untranslated region C > T gene polymorphism and cancer susceptibility--results of a meta-analysis. Asian Pac J Cancer Prev. 2012;13(7):3319-23.
