# Supplementary material for: Strong Cumulative Evidence of Associations of 6 Single Nucleotide Polymorphisms with Ovarian Cancer Risk: An Umbrella Review
Source: J Clin Med. 2023 Mar 3;12(5):2025. doi: 10.3390/jcm12052025 (PMC10004083; doi:10.3390/jcm12052025)
Supplement: Supplementary file 1 [file jcm-12-02025-s001.zip › jcm-2179460-supplementary/Supplementary File S3.pdf]

Supplementary File S3. No nominally statistically significant associations in the meta-analyses

SNPs	Gene name	Variant	Genetic model	No. of studies	Case	Control	Sample size	I^2 (95%CI)	OR (95% confidence interval)		*P* (Random effects)	95% prediction interval
									Fixed effects	Random effects		
rs4045402	*AR*	1 CAG_S; 2 CAG_L	1	6	1429	1785	3214	21.4 (0, 66)	1.05 (0.91, 1.22)	1.04 (0.87, 1.25)	0.675	0.70, 1.53
			2	6	1055	1255	2310	61.7 (7, 84)	1.24 (1.05, 1.48)	1.23 (0.88, 1.72)	0.228	0.47, 3.22
			3	9	6613	7041	13654	82.8 (69, 91)	0.78 (0.73, 0.84)	0.91 (0.72, 1.15)	0.445	0.45, 1.86
			4	6	1978	2393	4371	72.7 (37, 88)	1.18 (1.02, 1.36)	1.16 (0.84, 1.59)	0.363	0.43, 3.09
			5	6	3956	4786	8742	75.8 (46, 89)	1.11 (1.02, 1.21)	1.06 (0.87, 1.31)	0.560	0.56, 2.04
rs2273535	*AURKA*	1T; 2A	1	12	4039	6583	10622	43.2 (0, 71)	1.09 (1.01, 1.19)	1.09 (0.97, 1.22)	0.146	0.79, 1.49
			2	12	2758	4581	7339	10.8 (0, 50)	1.11 (0.93, 1.34)	1.11 (0.91, 1.36)	0.287	0.79, 1.56
			3	12	4249	6927	11176	54.5 (13, 76)	1.10 (1.01, 1.19)	1.09 (0.97, 1.24)	0.164	0.75, 1.59
			4	12	4249	6927	11176	0.0 (0, 58)	1.07 (0.89, 1.28)	1.07 (0.89, 1.28)	0.489	0.87, 1.31
			5	12	8498	13854	22352	57.8 (20, 78)	1.07 (1.01, 1.15)	1.07 (0.97, 1.19)	0.192	0.77, 1.49
rs144848	*BRCA2*	1N; 2H	1	7	7834	13449	21283	46.8 (0, 78)	1.02 (0.96, 1.07)	1.05 (0.95, 1.14)	0.343	0.82, 1.32
			2	7	5084	8707	13791	0.0 (0, 71)	1.07 (0.96, 1.18)	1.07 (0.96, 1.18)	0.239	0.93, 1.22
			3	7	8488	14510	22998	35.7 (0, 73)	1.02 (0.97, 1.08)	1.05 (0.97, 1.13)	0.243	0.87, 1.26
			4	7	8488	14510	22998	10.2 (0, 74)	1.06 (0.96, 1.17)	1.07 (0.95, 1.19)	0.273	0.87, 1.30
			5	7	16976	29020	45996	9.1 (0, 73)	1.02 (0.98, 1.07)	1.03 (0.98, 1.08)	0.227	0.95, 1.12
rs799917	*BRCA1*	1C; 2T	2	6	2156	3871	6027	0.0 (0, 75)	1.00 (0.84, 1.19)	1.00 (0.84, 1.19)	0.999	0.78, 1.29
rs1056836	*CYP1B1*	1G; 2C	1	8	833	1761	2594	0.0 (0, 68)	1.21 (0.98, 1.48)	1.21 (0.98, 1.48)	0.075	0.93, 1.56
			2	9	675	1456	2131	57.8 (12, 80)	1.11 (0.89, 1.38)	0.98 (0.67, 1.43)	0.903	0.33, 2.88
			3	9	1285	2659	3944	34.3 (0, 70)	1.15 (0.95, 1.39)	1.10 (0.84, 1.43)	0.483	0.59, 2.04
			4	9	1285	2659	3944	61.8 (21, 82)	0.97 (0.83, 1.13)	0.87 (0.65, 1.17)	0.368	0.38, 2.02
			5	9	2570	5318	7888	68.4 (37, 84)	1.03 (0.93, 1.14)	0.94 (0.76, 1.16)	0.559	0.49, 1.80
rs1056827	*CYP1B1*	1G; 2T	1	4	721	1055	1776	0.0 (0, 85)	1.08 (0.89, 1.31)	1.08 (0.89, 1.31)	0.431	0.71, 1.66
			2	3	454	673	1127	0.0 (0, 90)	1.10 (0.80, 1.52)	1.10 (0.80, 1.52)	0.552	0.14, 8.95
			3	4	799	1172	1971	0.0 (0, 85)	1.09 (0.90, 1.31)	1.09 (0.90, 1.31)	0.378	0.73, 1.63
			4	3	763	1119	1882	0.0 (0, 90)	1.07 (0.79, 1.46)	1.07 (0.79, 1.46)	0.668	0.14, 7.95
			5	4	1598	2344	3942	0.0 (0, 85)	1.07 (0.93, 1.22)	1.07 (0.93, 1.22)	0.380	0.78, 1.45
rs1800440	*CYP1B1*	1A; 2G	1	3	350	1523	1873	0.0 (0, 90)	1.24 (0.94, 1.63)	1.24 (0.94, 1.63)	0.136	0.20, 7.50
			2	3	249	1137	1386	0.0 (0, 90)	1.10 (0.52, 2.33)	1.10 (0.52, 2.33)	0.803	0.01, 140.71
			3	3	361	1577	1938	0.0 (0, 90)	1.21 (0.93, 1.59)	1.21 (0.93, 1.59)	0.161	0.21, 6.97
			4	3	361	1577	1938	0.0 (0, 90)	1.04 (0.50, 2.19)	1.04 (0.50, 2.19)	0.917	0.01, 128.51
			5	3	722	3154	3876	0.0 (0, 90)	1.15 (0.91, 1.45)	1.15 (0.91, 1.45)	0.240	0.25, 5.20
rs10012	*CYP1B1*	1Arg; 2Gly	1	4	754	1085	1839	11.5 (0, 86)	1.20 (0.96, 1.51)	1.25 (0.96, 1.64)	0.104	0.59, 2.67

			2	3	541	835	1376	0.0 (0, 90)	0.98 (0.64, 1.49)	0.98 (0.64, 1.49)	0.915	0.06, 14.89
			3	4	799	1169	1968	0.0 (0, 85)	1.16 (0.93, 1.44)	1.16 (0.93, 1.44)	0.181	0.72, 1.86
			4	3	763	1116	1879	0.0 (0, 90)	0.89 (0.60, 1.33)	0.89 (0.60, 1.33)	0.562	0.07, 11.88
			5	4	1598	2338	3936	0.0 (0, 85)	1.07 (0.90, 1.27)	1.07 (0.90, 1.27)	0.434	0.74, 1.55
			1	3	1699	2656	4355	56.0 (0, 87)	0.85 (0.75, 0.97)	0.80 (0.51, 1.25)	0.327	0.01, 102.51
			2	3	1260	1863	3123	79.0 (33, 93)	0.79 (0.61, 1.02)	1.25 (0.46, 3.35)	0.665	0.00, 110071.94
rs2066827	*CDKN1B*	1T; 2G	3	3	1829	2868	4697	68.7 (0, 91)	0.84 (0.74, 0.95)	0.88 (0.53, 1.46)	0.623	0.00, 259.19
			4	3	1829	2868	4697	80.8 (40, 94)	0.87 (0.68, 1.11)	1.21 (0.52, 2.85)	0.655	0.00, 20279.92
			5	3	3658	5736	9394	83.8 (51, 95)	0.87 (0.78, 0.96)	0.97 (0.59, 1.61)	0.904	0.00, 415.90
rs4646903	*CYP1A1*	1T; 2C	3	9	2495	3553	6048	0.0 (0, 65)	1.06 (0.93, 1.21)	1.06 (0.93, 1.21)	0.385	0.91, 1.24
rs1048943	*CYP1A1*	1 Ile; 2 Val	3	13	1815	3501	5316	73.5 (54, 85)	1.18 (0.97, 1.44)	1.14 (0.75, 1.73)	0.546	0.27, 4.76
			1	7	1043	2172	3215	0.0 (0, 71)	0.94 (0.80, 1.12)	0.94 (0.80, 1.12)	0.491	0.76, 1.18
			2	7	741	1489	2230	1.0 (0, 71)	1.00 (0.83, 1.22)	1.00 (0.83, 1.22)	0.986	0.77, 1.30
rs4680	*COMT*	1A; 2G	3	7	1439	2927	4366	0.0 (0, 71)	0.96 (0.82, 1.13)	0.96 (0.82, 1.13)	0.622	0.78, 1.18
			4	7	1439	2927	4366	0.0 (0, 71)	1.03 (0.88, 1.20)	1.03 (0.88, 1.20)	0.739	0.84, 1.26
			5	7	2878	5854	8732	0.8 (0, 71)	1.00 (0.91, 1.10)	1.00 (0.90, 1.10)	0.929	0.87, 1.13
			1	3	2669	3973	6642	0.0 (0, 90)	0.94 (0.84, 1.05)	0.94 (0.84, 1.05)	0.282	0.44, 1.99
rs11515	*CDKN2A*	1C; 2G	3	3	2737	4036	6773	0.0 (0, 90)	0.98 (0.87, 1.09)	0.98 (0.87, 1.09)	0.697	0.47, 2.03
			5	3	5474	8072	13546	0.0 (0, 90)	1.02 (0.93, 1.13)	1.02 (0.93, 1.13)	0.662	0.53, 1.96
			1	3	3139	5211	8350	0.0 (0, 90)	1.06 (0.94, 1.19)	1.06 (0.94, 1.19)	0.355	0.50, 2.23
			2	3	2593	4337	6930	0.0 (0, 90)	1.42 (0.87, 2.31)	1.42 (0.87, 2.31)	0.157	0.06, 33.38
rs3088440	*CDKN2A*	1C; 2T	3	3	3169	5248	8417	0.0 (0, 90)	1.07 (0.95, 1.20)	1.07 (0.95, 1.20)	0.253	0.51, 2.22
			4	3	3169	5248	8417	0.0 (0, 90)	1.41 (0.87, 2.29)	1.41 (0.87, 2.29)	0.169	0.06, 32.93
			5	3	6338	10496	16834	0.0 (0, 90)	1.07 (0.97, 1.19)	1.07 (0.97, 1.19)	0.184	0.54, 2.12
			2	8	2610	3380	5990	0.0 (0, 68)	1.11 (1.00, 1.23)	1.11 (1.00, 1.23)	0.060	0.97, 1.26
rs1271572	*ESR2*	1G; 2T	3	8	5109	6893	12002	0.0 (0, 68)	1.01 (0.93, 1.09)	1.01 (0.93, 1.09)	0.820	0.91, 1.11
			5	8	10218	13786	24004	89.3 (81, 94)	0.94 (0.90, 0.99)	1.02 (0.86, 1.22)	0.793	0.57, 1.85
rs3020450	*ESR2*	1G; 2A	2	4	673	694	1367	59.0 (0, 86)	0.83 (0.60, 1.15)	1.01 (0.57, 1.79)	0.976	0.10, 9.71
			4	4	1028	1182	2210	60.0 (0, 87)	0.96 (0.70, 1.31)	1.16 (0.66, 2.03)	0.599	0.13, 10.71
			1	9	892	2009	2901	77.9 (58, 88)	1.03 (0.85, 1.23)	1.21 (0.80, 1.84)	0.363	0.31, 4.76
rs13181	*ERCC2*	1A; 2C	4	9	1333	2691	4024	92.8 (88, 95)	2.64 (2.17, 3.22)	1.61 (0.70, 3.68)	0.261	0.09, 29.66
			5	9	2666	5382	8048	90.7 (85, 94)	1.66 (1.49, 1.86)	1.41 (0.96, 2.08)	0.079	0.35, 5.71
			1	3	300	919	1219	71.7 (4, 92)	0.75 (0.57, 0.99)	0.71 (0.42, 1.20)	0.201	0.00, 297.18
			2	3	227	599	826	58.2 (0, 88)	1.32 (0.85, 2.06)	1.20 (0.59, 2.45)	0.622	0.00, 2632.35
rs11615	*ERCC1*	1C; 2T	3	3	338	997	1335	79.9 (36, 94)	0.83 (0.64, 1.07)	0.78 (0.44, 1.39)	0.392	0.00, 776.89
			4	3	338	997	1335	30.9 (0, 93)	1.44 (0.94, 2.21)	1.39 (0.81, 2.36)	0.230	0.01, 174.37
			5	3	676	1994	2670	82.3 (46, 94)	0.97 (0.79, 1.18)	0.89 (0.55, 1.46)	0.652	0.00, 336.59
rs1051740	*EPHX1*	1T; 2C	1	5	1674	1580	3254	55.2 (0, 83)	0.99 (0.86, 1.13)	0.94 (0.73, 1.21)	0.650	0.44, 2.02

			2	5	1174	1123	2297	0.0 (0, 79)	1.08 (0.88, 1.34)	1.08 (0.88, 1.34)	0.456	0.77, 1.53
			3	5	1919	1829	3748	54.1 (0, 83)	1.01 (0.88, 1.15)	0.95 (0.76, 1.19)	0.658	0.48, 1.89
			4	5	1919	1829	3748	0.0 (0, 79)	1.08 (0.89, 1.32)	1.08 (0.89, 1.32)	0.452	0.78, 1.49
			5	5	3838	3658	7496	49.4 (0, 81)	1.02 (0.93, 1.13)	0.98 (0.83, 1.15)	0.759	0.61, 1.57
			1	5	392	1572	1964	0.0 (0, 79)	1.09 (0.86, 1.39)	1.09 (0.86, 1.39)	0.488	0.74, 1.61
			2	5	278	1052	1330	44.9 (0, 80)	1.19 (0.85, 1.68)	1.16 (0.71, 1.89)	0.556	0.28, 4.75
rs1799793	*ERCC2*	1G; 2A	3	5	492	1841	2333	0.0 (0, 79)	1.11 (0.88, 1.39)	1.11 (0.88, 1.39)	0.388	0.76, 1.60
			4	5	492	1841	2333	59.7 (0, 85)	1.08 (0.80, 1.46)	1.03 (0.63, 1.69)	0.906	0.21, 4.98
			5	5	984	3682	4666	39.9 (0, 78)	1.06 (0.91, 1.25)	1.06 (0.86, 1.30)	0.610	0.59, 1.90
			1	3	365	718	1083	73.5 (11, 92)	1.07 (0.81, 1.41)	1.08 (0.60, 1.93)	0.802	0.00, 904.03
			2	3	367	480	847	89.7 (72, 96)	2.25 (1.66, 3.04)	1.70 (0.59, 4.89)	0.323	0.00, 840570.19
rs238406	*ERCC2*	1C; 2A	3	3	607	943	1550	86.8 (62, 95)	1.46 (1.13, 1.88)	1.33 (0.61, 2.88)	0.477	0.00, 18030.39
			4	3	607	943	1550	88.2 (67, 96)	2.09 (1.65, 2.64)	1.61 (0.75, 3.45)	0.221	0.00, 19570.09
			5	3	1214	1886	3100	92.4 (81, 97)	1.58 (1.35, 1.83)	1.36 (0.75, 2.47)	0.311	0.00, 2462.42
			1	4	474	1556	2030	94.1 (88, 97)	1.13 (0.89, 1.43)	1.37 (0.48, 3.87)	0.557	0.01, 198.89
			2	4	316	1278	1594	90.4 (79, 96)	1.05 (0.72, 1.54)	1.96 (0.52, 7.37)	0.318	0.00, 946.78
rs1695	*GSTP1*	1A; 2G	3	4	534	1646	2180	95.3 (91, 98)	1.15 (0.92, 1.44)	1.46 (0.49, 4.35)	0.502	0.01, 281.28
			4	4	534	1646	2180	87.8 (71, 95)	1.09 (0.77, 1.56)	1.88 (0.61, 5.76)	0.271	0.01, 327.28
			5	4	1068	3292	4360	96.4 (93, 98)	1.14 (0.96, 1.35)	1.51 (0.58, 3.92)	0.396	0.02, 149.94
			2	4	263	417	680	65.5 (0, 88)	4.46 (1.95, 10.23)	3.46 (0.73, 16.33)	0.117	0.01, 1965.81
rs1801200	*HER2*	1I; 2V	3	4	348	540	888	68.8 (10, 89)	1.20 (0.88, 1.64)	1.15 (0.64, 2.07)	0.647	0.10, 13.52
			4	4	348	540	888	62.7 (0, 87)	4.61 (2.02, 10.50)	3.69 (0.84, 16.23)	0.084	0.01, 1435.33
			5	4	696	1080	1776	92.5 (84, 96)	1.01 (0.78, 1.32)	1.03 (0.38, 2.82)	0.958	0.01, 121.29
			1	4	1056	1858	2914	0.0 (0, 85)	1.17 (0.94, 1.46)	1.17 (0.94, 1.46)	0.166	0.72, 1.90
rs1466445	*ITGA1*	19A; 26A	2	3	760	1366	2126	46.8 (0, 84)	2.46 (1.07, 5.66)	3.26 (0.85, 12.51)	0.086	0.00, 2436300.00
			3	4	1073	1866	2939	0.0 (0, 85)	1.22 (0.98, 1.51)	1.22 (0.98, 1.51)	0.072	0.76, 1.95
			4	3	929	1587	2516	45.3 (0, 84)	2.41 (1.05, 5.53)	3.12 (0.83, 11.71)	0.091	0.00, 1640495.63
rs61764370	*KRAS*	1T; 2G	3	21	9169	10435	19604	48.0 (14, 69)	1.05 (0.97, 1.13)	1.06 (0.95, 1.19)	0.228	0.73, 1.55
			1	12	3728	4119	7847	21.5 (0, 60)	1.07 (0.97, 1.17)	1.08 (0.96, 1.21)	0.228	0.84, 1.37
			2	11	2356	2615	4971	66.5 (37, 82)	1.15 (0.99, 1.33)	1.25 (0.92, 1.71)	0.150	0.49, 3.18
rs1801133	*MTHFR*	1C; 2T	3	12	4214	4591	8805	43.7 (0, 71)	1.08 (0.99, 1.18)	1.11 (0.96, 1.27)	0.156	0.77, 1.59
			4	11	4199	4566	8765	59.8 (22, 79)	1.11 (0.96, 1.27)	1.18 (0.91, 1.55)	0.216	0.55, 2.55
			5	12	8428	9182	17610	60.8 (26, 79)	1.07 (1.00, 1.14)	1.11 (0.97, 1.25)	0.121	0.76, 1.60
			1	7	3189	3405	6594	0.0 (0, 71)	0.97 (0.88, 1.07)	0.97 (0.88, 1.07)	0.553	0.85, 1.10
			2	7	2066	2144	4210	14.4 (0, 58)	1.07 (0.91, 1.26)	1.08 (0.89, 1.31)	0.441	0.75, 1.55
rs1801131	*MTHFR*	1A; 2C	3	7	3553	3762	7315	0.0 (0, 71)	0.99 (0.90, 1.09)	0.99 (0.90, 1.09)	0.844	0.88, 1.12
			4	7	3553	3762	7315	26.3 (0, 68)	1.09 (0.93, 1.27)	1.10 (0.90, 1.36)	0.361	0.70, 1.73
			5	7	7106	7524	14630	1.7 (0, 71)	1.01 (0.94, 1.09)	1.01 (0.94, 1.09)	0.702	0.92, 1.12

rs1799750	*MMP1*	1:2G; 2:1G	1	5	609	953	1562	52.3 (0, 82)	1.04 (0.83, 1.29)	1.09 (0.78, 1.54)	0.606	0.39, 3.05
			2	5	404	634	1038	0.0 (0, 79)	0.93 (0.70, 1.23)	0.93 (0.70, 1.23)	0.598	0.58, 1.47
			3	5	754	1184	1938	23.2 (0, 68)	1.01 (0.82, 1.24)	1.02 (0.80, 1.31)	0.855	0.57, 1.83
			4	5	754	1184	1938	16.6 (0, 83)	0.95 (0.75, 1.22)	0.93 (0.70, 1.24)	0.635	0.50, 1.75
			5	5	1158	1818	2976	43.2 (0, 79)	1.00 (0.85, 1.17)	1.03 (0.82, 1.29)	0.798	0.54, 1.96
rs34093618	*MMP3*	1:6A; 2:5A	1	3	221	353	574	68.2 (0, 91)	0.88 (0.60, 1.29)	1.08 (0.51, 2.30)	0.837	0.00, 4745.40
			2	3	166	246	412	0.0 (0, 90)	1.25 (0.69, 2.24)	1.25 (0.69, 2.24)	0.463	0.03, 56.49
			3	3	265	425	690	51.6 (0, 86)	0.94 (0.65, 1.35)	1.04 (0.59, 1.85)	0.894	0.00, 373.69
			4	3	265	425	690	40.7 (0, 82)	1.19 (0.72, 1.95)	1.03 (0.48, 2.22)	0.941	0.00, 1988.11
			5	3	530	850	1380	0.0 (0, 90)	1.01 (0.79, 1.31)	1.01 (0.79, 1.31)	0.917	0.19, 5.28
rs35068180	*MMP3*	15A; 26A	1	3	143	251	394	66.5 (0, 90)	0.85 (0.50, 1.44)	1.06 (0.36, 3.18)	0.915	0.00, 225129.72
			2	3	166	246	412	0.0 (0, 90)	0.80 (0.45, 1.44)	0.80 (0.45, 1.44)	0.456	0.02, 35.11
			3	3	265	425	690	42.3 (0, 83)	0.85 (0.52, 1.40)	0.98 (0.45, 2.15)	0.967	0.00, 2268.01
			4	3	265	425	690	52.2 (0, 86)	1.07 (0.75, 1.54)	0.96 (0.54, 1.71)	0.895	0.00, 362.01
			5	3	530	850	1380	0.0 (0, 90)	0.99 (0.76, 1.27)	0.99 (0.76, 1.27)	0.910	0.19, 5.11
rs11614913	miR-196a2	1C; 2T	1	4	682	753	1435	55.7 (0, 85)	0.94 (0.74, 1.19)	0.99 (0.67, 1.44)	0.941	0.22, 4.38
			2	4	428	556	984	51.5 (0, 84)	0.69 (0.53, 0.90)	0.75 (0.50, 1.12)	0.161	0.16, 3.48
			3	4	909	1100	2009	60.4 (0, 87)	0.84 (0.67, 1.05)	0.90 (0.61, 1.32)	0.593	0.19, 4.20
			5	4	1818	2200	4018	43.7 (0, 81)	0.83 (0.73, 0.94)	0.86 (0.72, 1.02)	0.089	0.45, 1.64
rs2279744	*MDM2*	1T; 2G	1	3	440	460	900	64.8 (0, 90)	0.94 (0.71, 1.24)	0.90 (0.55, 1.47)	0.671	0.00, 207.90
			2	3	313	315	628	83.9 (52, 95)	0.97 (0.71, 1.34)	0.95 (0.41, 2.18)	0.897	0.00, 22420.91
			3	3	599	622	1221	79.6 (35, 94)	0.95 (0.73, 1.23)	0.90 (0.49, 1.66)	0.745	0.00, 1244.56
			4	3	599	622	1221	74.7 (16, 92)	1.02 (0.79, 1.31)	1.03 (0.60, 1.77)	0.911	0.00, 542.06
			5	3	1198	1244	2442	84.8 (55, 95)	0.99 (0.84, 1.16)	0.98 (0.64, 1.5)	0.909	0.01, 175.08
rs1052133	*OGG1*	1C; 2G	2	3	871	809	1680	86.8 (62, 95)	1.88 (1.53, 2.31)	1.60 (0.8, 3.21)	0.189	0.00, 5916.97
			3	3	1231	1617	2848	69.5 (0, 91)	1.07 (0.90, 1.29)	0.98 (0.67, 1.42)	0.912	0.02, 63.69
			4	3	1231	1617	2848	93.1 (83, 97)	2.41 (2.04, 2.83)	2.06 (0.97, 4.37)	0.060	0.00, 17730.04
			5	3	2462	3234	5696	93.7 (85, 97)	1.55 (1.39, 1.73)	1.30 (0.79, 2.16)	0.303	0.00, 656.07
rs10895068	*PGR*	1G; 2A	1	8	2838	4293	7131	45.4 (0, 76)	1.03 (0.87, 1.22)	1.02 (0.80, 1.31)	0.869	0.54, 1.94
			2	8	2560	3886	6446	0.0 (0, 68)	1.61 (0.64, 4.03)	1.61 (0.64, 4.03)	0.314	0.51, 5.07
			3	8	2848	4305	7153	42.8 (0, 75)	1.04 (0.88, 1.22)	1.03 (0.81, 1.30)	0.823	0.56, 1.89
			4	8	2848	4305	7153	0.0 (0, 68)	1.61 (0.64, 4.04)	1.61 (0.64, 4.04)	0.313	0.51, 5.07
			5	8	5696	8610	14306	37.4 (0, 72)	1.04 (0.89, 1.22)	1.03 (0.83, 1.29)	0.762	0.61, 1.76
rs1042838	*PGR*	1G; 2T	1	5	1948	2220	4168	78.9 (50, 91)	0.87 (0.76, 1.00)	1.00 (0.72, 1.40)	0.999	0.30, 3.28
			2	5	1484	1632	3116	54.5 (0, 83)	1.22 (0.80, 1.85)	1.57 (0.74, 3.35)	0.242	0.16, 15.44
			3	7	2205	3222	5427	86.5 (74, 93)	0.99 (0.87, 1.13)	1.31 (0.87, 1.98)	0.194	0.34, 5.07
			4	5	2007	2270	4277	49.0 (0, 81)	1.28 (0.85, 1.94)	1.57 (0.77, 3.19)	0.211	0.20, 12.39
			5	5	4014	4540	8554	79.0 (50, 91)	0.95 (0.84, 1.07)	1.08 (0.81, 1.44)	0.584	0.39, 2.99

rs1801282	*PPARG*	1C; 2G	1	5	1366	2108	3474	0.0 (0, 79)	1.03 (0.87, 1.23)	1.03 (0.87, 1.23)	0.733	0.78, 1.36
			2	4	1069	1650	2719	0.0 (0, 85)	1.05 (0.56, 1.98)	1.05 (0.56, 1.98)	0.886	0.26, 4.22
			3	5	1384	2137	3521	0.0 (0, 79)	1.03 (0.87, 1.21)	1.03 (0.87, 1.21)	0.773	0.78, 1.35
			4	4	1358	2057	3415	0.0 (0, 85)	1.04 (0.55, 1.97)	1.04 (0.55, 1.97)	0.893	0.26, 4.20
			5	5	2768	4274	7042	0.0 (0, 79)	1.02 (0.87, 1.19)	1.02 (0.87, 1.19)	0.827	0.79, 1.31
rs6917	*PHB*	1C; 2T	1	3	783	533	1316	0.0 (0, 90)	1.03 (0.81, 1.32)	1.03 (0.81, 1.32)	0.816	0.21, 5.09
			2	3	574	396	970	29.5 (0, 93)	1.06 (0.51, 2.19)	0.96 (0.33, 2.74)	0.933	0.00, 15057.63
			3	3	806	547	1353	0.0 (0, 90)	1.03 (0.81, 1.31)	1.03 (0.81, 1.31)	0.800	0.22, 4.87
			4	3	806	547	1353	33.4 (0, 93)	1.05 (0.51, 2.16)	0.94 (0.31, 2.80)	0.906	0.00, 27515.47
			5	3	1612	1094	2706	0.0 (0, 90)	1.03 (0.83, 1.26)	1.03 (0.83, 1.26)	0.813	0.26, 3.98
rs1801320	*RAD51*	1G; 2C	1	3	2162	3911	6073	0.0 (0, 90)	1.04 (0.89, 1.22)	1.04 (0.89, 1.22)	0.601	0.38, 2.87
			2	3	1889	3431	5320	2.3 (0, 90)	1.26 (0.61, 2.60)	1.26 (0.60, 2.64)	0.540	0.01, 189.22
			3	6	2388	4411	6799	39.8 (0, 76)	1.00 (0.87, 1.16)	0.98 (0.76, 1.27)	0.886	0.52, 1.85
			4	3	2175	3931	6106	5.2 (0, 90)	1.25 (0.61, 2.58)	1.25 (0.58, 2.67)	0.571	0.01, 276.33
			5	3	4350	7862	12212	0.0 (0, 90)	1.05 (0.91, 1.22)	1.05 (0.91, 1.22)	0.494	0.41, 2.69
rs11466445	*TGFBR1*	19A; 26A	1	4	1056	1858	2914	0.0 (0, 85)	1.17 (0.94, 1.46)	1.17 (0.94, 1.46)	0.166	0.72, 1.90
			2	3	760	1366	2126	46.8 (0, 84)	2.46 (1.07, 5.66)	3.26 (0.85, 12.51)	0.086	0.00, 2436300.00
			3	4	1073	1866	2939	0.0 (0, 85)	1.22 (0.98, 1.51)	1.22 (0.98, 1.51)	0.072	0.76, 1.95
			4	3	929	1587	2516	45.3 (0, 84)	2.41 (1.05, 5.53)	3.12 (0.83, 11.71)	0.091	0.00, 1640495.63
rs1042522	*TP53*	1Arg; 2Pro	1	24	2958	6166	9124	61.9 (41, 76)	1.12 (1.02, 1.24)	1.14 (0.96, 1.36)	0.127	0.59, 2.23
			2	23	1909	4181	6090	43.2 (7, 65)	1.14 (0.96, 1.34)	1.04 (0.81, 1.34)	0.747	0.46, 2.34
			3	24	3271	6842	10113	65.0 (46, 77)	1.12 (1.03, 1.23)	1.12 (0.94, 1.33)	0.197	0.57, 2.20
			4	23	3258	6829	10087	24.8 (0, 55)	1.09 (0.93, 1.28)	1.04 (0.86, 1.27)	0.684	0.62, 1.74
			5	24	6542	13684	20226	62.1 (41, 76)	1.08 (1.01, 1.16)	1.06 (0.93, 1.20)	0.372	0.65, 1.72
rs2228570	*VDR*	1C; 2T	4	14	4448	7242	11690	7.4 (0, 44)	1.11 (1.00, 1.23)	1.11 (0.99, 1.24)	0.072	0.93, 1.33
rs1544410	*VDR*	1b; 2B	5	4	599	759	1358	0.0 (0, 85)	1.08 (0.93, 1.25)	1.08 (0.93, 1.25)	0.295	0.78, 1.49
rs11568820	*VDR*	1G; 2A	2	3	963	1435	2398	0.0 (0, 90)	1.07 (0.78, 1.48)	1.07 (0.78, 1.48)	0.678	0.13, 8.73
			4	3	1475	2055	3530	0.0 (0, 90)	1.00 (0.73, 1.38)	1.00 (0.73, 1.38)	0.982	0.13, 7.97
rs3025039	*VEGFA*	1C; 2T	1	7	1281	1647	2928	84.5 (70, 92)	1.07 (0.89, 1.27)	1.07 (0.65, 1.76)	0.785	0.21, 5.47
			2	7	1010	1329	2339	0.0 (0, 71)	1.14 (0.68, 1.92)	1.14 (0.68, 1.92)	0.609	0.58, 2.25
			3	7	1345	1751	3096	84.7 (70, 92)	1.08 (0.91, 1.28)	1.12 (0.69, 1.82)	0.649	0.23, 5.49
			4	7	1345	1751	3096	0.0 (0, 71)	1.18 (0.77, 1.81)	1.18 (0.77, 1.81)	0.442	0.68, 2.07
			5	7	2690	3502	6192	83.8 (68, 92)	1.08 (0.93, 1.25)	1.17 (0.79, 1.72)	0.435	0.32, 4.31
rs833061	*VEGFA*	1C; 2T	1	4	455	601	1056	42.7 (0, 81)	1.67 (1.26, 2.22)	1.43 (0.90, 2.26)	0.128	0.27, 7.44
rs699947	*VEGFA*	1C; 2A	1	4	928	1129	2057	55.3 (0, 85)	1.23 (1.02, 1.47)	1.25 (0.95, 1.64)	0.107	0.43, 3.62
			2	4	606	790	1396	66.8 (3, 89)	1.18 (0.86, 1.62)	1.33 (0.75, 2.35)	0.332	0.12, 14.40
			3	4	1022	1228	2250	71.3 (18, 90)	1.23 (1.04, 1.46)	1.27 (0.92, 1.76)	0.139	0.32, 5.07
			4	4	1022	1228	2250	56.2 (0, 85)	1.11 (0.82, 1.50)	1.21 (0.75, 1.96)	0.434	0.18, 7.97

			5	4	2044	2456	4500	79.6 (46, 92)	1.16 (1.02, 1.33)	1.23 (0.91, 1.66)	0.179	0.32, 4.68
rs731236	*VDR*	1t; 2T	5	3	237	446	683	0.0 (0, 90)	0.94 (0.72, 1.21)	0.94 (0.72, 1.21)	0.619	0.17, 5.02
rs861539	*XRCC3*	1C; 2T	1	6	3618	6749	10367	0.0 (0, 75)	0.92 (0.85, 1.00)	0.92 (0.85, 1.00)	0.060	0.82, 1.04
			2	6	2342	4226	6568	0.0 (0, 75)	0.94 (0.84, 1.06)	0.94 (0.84, 1.06)	0.308	0.80, 1.11
			3	6	4283	7925	12208	0.0 (0, 75)	0.93 (0.86, 1.01)	0.93 (0.86, 1.01)	0.069	0.83, 1.04
			4	6	4283	7925	12208	0.0 (0, 75)	0.98 (0.88, 1.09)	0.98 (0.88, 1.09)	0.747	0.85, 1.14
			5	6	8566	15850	24416	0.0 (0, 75)	0.96 (0.91, 1.02)	0.96 (0.91, 1.02)	0.150	0.89, 1.04
rs1799794	*XRCC3*	1A; 2G	1	4	2063	4359	6422	0.0 (0, 85)	1.09 (0.97, 1.22)	1.09 (0.97, 1.22)	0.145	0.85, 1.40
			3	4	2137	4552	6689	0.0 (0, 85)	1.05 (0.94, 1.17)	1.05 (0.94, 1.17)	0.436	0.82, 1.34
			5	4	4274	9104	13378	0.0 (0, 85)	0.99 (0.90, 1.09)	0.99 (0.90, 1.09)	0.854	0.80, 1.22
rs3218536	*XRCC2*	1G; 2A	2	8	4218	7092	11310	3.1 (0, 69)	0.65 (0.40, 1.06)	0.65 (0.39, 1.06)	0.085	0.32, 1.30
			4	8	4858	8310	13168	3.3 (0, 69)	0.66 (0.41, 1.08)	0.66 (0.40, 1.08)	0.098	0.32, 1.33

CI, confidence interval; SNPs, single nucleotide polymorphisms.
